# Preferential *MGMT* methylation could predispose a subset of *KIT/PDGFRA*-WT GISTs, including SDH-deficient ones, to respond to alkylating agents

**DOI:** 10.1186/s13148-018-0594-9

**Published:** 2019-01-07

**Authors:** Riccardo Ricci, Maurizio Martini, Gloria Ravegnini, Tonia Cenci, Massimo Milione, Paola Lanza, Francesco Pierconti, Donatella Santini, Sabrina Angelini, Alberto Biondi, Fausto Rosa, Sergio Alfieri, Gennaro Clemente, Roberto Persiani, Alessandra Cassano, Maria A. Pantaleo, Luigi M. Larocca

**Affiliations:** 1 Department of Pathology, Università Cattolica del Sacro Cuore, Largo F.Vito 1, 00168 Rome, Italy; 2 UOC di Anatomia Patologica, Fondazione Policlinico Universitario “A. Gemelli” IRCCS, Largo A. Gemelli 8, 00168 Rome, Italy; 3 Department of Pharmacy and Biotechnology, University of Bologna, via Massarenti 9, 40138 Bologna, Italy; 4 Department of Pathology and Laboratory Medicine, Fondazione IRCCS Istituto Nazionale dei Tumori, Via Venezian, 20100 Milan, Italy; 5 Pathology Unit, S.Orsola-Malpighi Hospital, University of Bologna, via Massarenti 9, 40138 Bologna, Italy; 6 UOC di Chirurgia Generale, Fondazione Policlinico Universitario “A. Gemelli” IRCCS, Largo A. Gemelli 8, 00168 Rome, Italy; 7 UOC di Chirurgia Digestiva, Fondazione Policlinico Universitario “A. Gemelli” IRCCS, Largo A. Gemelli 8, 00168 Rome, Italy; 8 Department of Surgery, Università Cattolica del Sacro Cuore, Largo F. Vito 1, 00168 Rome, Italy; 9 UOC di Chirurgia Generale ed Epato-Biliare, Fondazione Policlinico Universitario “A. Gemelli” IRCCS, Largo A. Gemelli 8, 00168 Rome, Italy; 10 Department of Internal Medicine, Università Cattolica del Sacro Cuore, Largo F. Vito 1, 00168 Rome, Italy; 11 UOC di Oncologia Medica, Fondazione Policlinico Universitario “A. Gemelli” IRCCS, Largo A. Gemelli 8, 00168 Rome, Italy; 12 Department of Experimental, Diagnostic and Specialty Medicine, University of Bologna, via Massarenti 9, 40138 Bologna, Italy

**Keywords:** Gastrointestinal stromal tumors, Succinate dehydrogenase, *O*^*6*^*-methylguanine DNA methyltransferase*, DNA methylation, Wild-type GIST, Molecular diagnosis

## Abstract

**Background:**

Succinate dehydrogenase (SDH)-deficient gastrointestinal stromal tumors (GISTs) constitute a small *KIT*/*PDGFRA*-WT GIST subgroup featuring DNA methylation which, although pervasive, appears nevertheless not randomly distributed. Although often indolent, these tumors are mostly chemorefractory in aggressive cases. Promoter methylation-induced *O*^*6*^*-methylguanine DNA methyltransferase* (*MGMT*) inactivation improves the efficacy of alkylating agents in gliomas, colorectal cancer and diffuse large B cell lymphoma. *MGMT* methylation has been found in some GISTs, without determining SDH status. Thirty-six GISTs were enrolled in past sarcoma trials testing alkylating agents, with negative results. Nevertheless, a possible effect on *MGMT*-methylated GISTs could have escaped detection, since tested GISTs were neither selected by genotype nor investigated for SDH; MGMT was studied in two cases only, revealing baseline activity; these trials were performed prior to the adoption of Choi criteria, the most sensitive for detecting GIST responses to therapy. Under these circumstances, we investigated whether *MGMT* methylation is preferentially found in SDH-deficient cases (identified by SDHB immunohistochemistry) by analyzing 48 pathogenetically heterogeneous GISTs by methylation-specific PCR, as a premise for possible investigations on the use of alkylating drugs in these tumors.

**Results:**

Nine GISTs of our series were SDH-deficient, revealing significantly enriched in *MGMT*-methylated cases (6/9–67%–, vs. 6/39–15%– of SDH-proficient GISTs; *p* = 0.004). The pathogenetically heterogeneous *KIT/PDGFRA*-WT GISTs were also significantly *MGMT*-methylated (11/24–46%–, vs. 1/24–4%– of *KIT/PDGFRA*-mutant cases, *p* = 0.002).

**Conclusions:**

A subset of *KIT/PDGFRA*-WT GISTs, including their largest pathogenetically characterized subgroup (i.e., SDH-deficient ones), is preferentially *MGMT*-methylated. This finding could foster a reappraisal of alkylating agents for treating malignant cases occurring among these overall chemorefractory tumors.

**Electronic supplementary material:**

The online version of this article (10.1186/s13148-018-0594-9) contains supplementary material, which is available to authorized users.

## Background

Gastrointestinal (GI) stromal tumors (GISTs), the most common mesenchymal tumors of the GI tract, revealed a heterogeneous family of tumors differing in molecular trigger and, consequently, in pathogenesis, prognosis and therapy [1, 2]. Their vast majority hinges upon activating mutations of either *KIT* or *platelet-derived growth factor receptor alpha* (*PDGFRA*), being often responsive to the tyrosin-kinase (TK)-inhibitor (TKI) imatinib. GISTs wild-type (WT) for these two genes, including their largest pathogenetically characterized subgroup (i.e., the succinate dehydrogenase (SDH)-deficient subset), although often indolent, may behave aggressively, being usually unresponsive to the TKI therapies commonly used [3–5]. In particular, presently, there is no specific evidence of an effective therapy for SDH-deficient GISTs with the possible exception of regorafenib and sunitinib for their anti-angiogenic mechanism of action, even though mature data are still lacking [6, 7].

The exploitation of reduced activity of O^6^-methylguanine DNA methyltransferase (MGMT) due to epigenetic silencing is a promising approach for selectively targeting tumors employing alkylating agents, with reduced host toxicity. This strategy has been shown to be effective in glioma, colorectal cancer and diffuse large B cell lymphoma [8–11].

SDH-deficient GISTs feature pervasive DNA methylation, which appears, nevertheless, to be not randomly distributed, as evidenced by the significant number of recurrent hypo- and hypermethylated genomic targets found in these tumors and by the hypermethylation of *SDHC*, typical of SDH-deficient GISTs WT for *SDH-A*, *B*, *C*, and *D* genes (collectively termed *SDHx*) but not of *SDHx*-mutant ones [12–14]. Noticeably, although reported in a few GISTs [15–17], *MGMT* methylation has never been investigated in SDH-deficient cases. Trials on the use of alkylating agents temozolomide and carmustine in sarcomas evaluated 36 GISTs, with negative results [18–20]. However, these GISTs were neither selected by genotype nor, coherent with the epoch of the studies, selected and/or investigated for SDH. Moreover, MGMT was studied in two cases only, revealing baseline activity; the actual MGMT depletion following administration of O^6^-benzylguanine, a MGMT-inactivating substrate, was not verified [20]. Finally, these trials were performed prior to the adoption of Choi criteria, the most sensitive method for assessing GIST response to therapy [21]. A possible effect of alkylating agents on *MGMT*-methylated GISTs could thus have been missed, possibly due to an inappropriate selection of cases and a lack of proper evaluation of tumor response. Following these premises, we analyzed whether SDH-deficient GISTs are preferentially *MGMT*-methylated, as a precondition for possible investigations on the use of alkylating drugs in the treatment of these tumors.

## Methods

### Study population

Forty-eight GISTs, assorted so as to represent various pathogenetic types, were retrieved from the files of the pathology departments of the Catholic University of Rome and of the Sant’Orsola-Malpighi Hospital of Bologna, differing in anatomic site and morphology. All the cases had been previously characterized by hematoxylin/eosin staining of sections from formalin-fixed, paraffin-embedded (FFPE) specimens; by immunohistochemistry (IHC) for CD117 and DOG1; and by genetic analysis of *KIT* (exons 9, 11, 13, and 17) and *PDGFRA* (exons 12, 14, and 18). In cases WT for these genes, *KRAS* (exon 2) and *BRAF* (V600E) were also analyzed; previously reported protocols were followed [22–24]. *BRAF* status was also analyzed in five *KIT* or *PDGFRA*-mutant cases (noticeably, *BRAF* mutations have been only exceptionally detected together with *KIT* or *PDGFRA* ones [25]). Five GISTs (from three patients) arose in the context of neurofibromatosis type 1 (NF1). Tumor features are detailed in Table 1. Four cases, including two tumors previously characterized for SDH status, have been previously published [22, 26, 27].

**Table 1:** Clinicopathologic and genetic features of the investigated GISTs

GIST no.	Age at diagnosis (years)	Sex	Site	Morphology	Genotype					NF1	SDHB IHC	*MGMT* methylation status	MGMT IHC score
					*KIT*	*PDGFRA*	*BRAF*	*KRAS*	*SDHx*				
1	64	F	Stomach	S^a^	Exon 11 p.E554_K558 (c.1660_1674del15)	WT^b^	NA^c^	NA	NA	N^d^	NA	U^e^	NA
2	86	F	Stomach	M	Exon 11 p.H580_K581ins9 (c.1740_1741ins27)	WT	NA	NA	NA	N	POS^f^	U	NA
3	67	F	Stomach	S	Exon 11 p.W557>G (c. 1669 T>G)	WT	WT	NA	NA	N	POS	U	3
4	91	F	Stomach	S	Exon 11 W557_K558del (c.1669_1674delTGGAAG)	WT	WT	NA	NA	N	POS	U	3
5	72	M	Stomach	S	Exon 11 W557R (c.1669 T>A)	WT	WT	NA	NA	N	POS	U	2
6	68	M	Stomach	S	Exon 11 p.V555_Q556del (c.1663_1668delGTACAG)	WT	NA	NA	NA	N	NA	U	NA
7	63	F	Stomach	M	Exon 11 p.D579_W582del12 (c.1735_1746del12)	WT	NA	NA	NA	N	NA	U	NA
8	74	M	Stomach	S	Exon 11 p.N566_Q575del (c.1696_1725del30)	WT	NA	NA	NA	N	NA	U	NA
9	49	F	Stomach	S	Exon 11 p.V559D (c.1676 T>A)	WT	NA	NA	NA	N	POS	M	1
10	39	F	Ileum	M	Exon 11 p.V560D (c.1679 T>A)	WT	NA	NA	NA	N	NA	U	NA
11	80	F	Jejunum	S	Exon 11 p.W557_K558del (c.1669_1674delTGGAAG)	WT	NA	NA	NA	N	NA	U	NA
12	51	M	Ileum	S	Exon 11 p.M552_W557 del (c.1653_1670del18)	WT	NA	NA	NA	N	POS	U	3
13	64	F	Jejunum	S	Exon 9 p.Y503_F504insYA (c.1509_1510insGCCTAT)	WT	NA	NA	NA	N	NA	U	NA
14	67	F	Stomach	S	Exon 9 p.Y503_F504insYA (c.1509_1510insGCCTAT)	WT	NA	NA	NA	N	NA	U	NA
15	47	M	Stomach	E	WT	Exon 18 p.D842V (c.2525A>T)	NA	NA	NA	N	NA	U	NA
16	74	M	Stomach	M	WT	Exon 18 p.D842V (c.2525A>T)	WT	NA	NA	N	NA	U	NA
17	57	F	Stomach	S	WT	Exon 18 p.D842V (c.2525A>T)	NA	NA	NA	N	POS	U	2
18	62	M	Jejunum	M	WT	Exon 18 p.D842V (c.2525A>T)	WT	NA	NA	N	POS	U	3
19	53	M	Stomach	E	WT	Exon 18 p.I843_D846delIMHD (c.2527_2538del12)	NA	NA	NA	N	NA	U	NA
20	77	F	Stomach	M	WT	Exon 18 p.D842V (c.2525A>T)	NA	NA	NA	N	NA	U	NA
21^h^	66	M	Stomach (multifocal)	E	WT	Exon 14 p.P653L (c.1957_1958CC>TT) germline	NA	NA	NA	N	POS	U	2
22^i^	67	M	Jejunum	S	Exon 11 p.Q556_W557delinsR (c.1667_1669delAGT) somatic	Exon 14 p.P653L (c.1957_1958CC>TT) germline	NA	NA	NA	N	NA	U	NA
23	78	M	Stomach	E	WT	Exon 12 p.V561D (c.1682 T>A)	NA	NA	NA	N	NA	U	NA
24	42	M	Stomach	E	WT	Exon 12 p.V561D (c.1682 T>A)	NA	NA	NA	N	POS	U	NA
25	68	M	Stomach	M	WT	WT	WT	WT	NA	N	POS	U	1
26	41	F	Pericolic	M	WT	WT	WT	WT	NA	N	POS	U	3
27	61	F	Jejunum	S	WT	WT	WT	WT	NA	N	POS	U	3
28	52	M	Duodenum-Jejunum	S	WT	WT	WT	WT	NA	N	POS	M	1
29	46	F	Small bowel NOS^g^	S	WT	WT	WT	WT	NA	Y	POS	U	2
30	67	F	Duodenum	S	WT	WT	WT	WT	NA	N	POS	U	3
31	73	M	Ileum	S	WT	WT	WT	WT	NA	N	POS	M	1
32	73	F	Stomach	S	WT	WT	WT	WT	NA	N	POS	M	1
33	48	M	Stomach	S	WT	WT	WT	WT	NA	Y	POS	M	3
34	64	F	Jejunum	S	WT	WT	WT	WT	NA	Y	POS	U	NA
35			Jejunum	S	WT	WT	WT	WT	NA		POS	U	NA
36			Jejunum	S	WT	WT	WT	WT	NA		POS	U	NA
37	57	M	Duodenum	S	WT	WT	WT	WT	NA	N	POS	U	3
38	65	F	Ileum	S	WT	WT	WT	WT	NA	N	POS	U	3
39	34	M	Stomach	E	WT	WT	WT	WT	NA	N	POS	M	NA
40 ^j^	28	F	Stomach	M	WT	WT	WT	WT	*SDHA* Exon 9 p.S384X (c.1151C>G) heterozygous germline and homozygous somatic	N	NEG	M	NA
41 ^j^	30	M	Stomach	M	WT	WT	WT	WT	*SDHA* Exon 2 p.R31X (c.91C>T) heterozygous germline and Exon 13 p.R589W (c.1765C>T) heterozygous somatic	N	NEG	M	NA
42	21	F	Stomach	S	WT	WT	WT	WT	*SDHA* Exon5 p.R171H (c.G512G>A) heterozygous germline and homozygous somatic	N	NEG	U	NA
43	60	F	Stomach (multifocal)^k^	M	WT	WT	WT	WT	*SDHA* Exon1 p.R9Q (c.26G>A) heterozygous somatic and Exon 13 p.Q577K (c.1729 C>A) heterozygous somatic	N	NEG	U	3
44	48	F	Stomach	E	WT	WT	WT	WT	*SDHA* Exon9 p.G419R (c.1255G>A) heterozygous somatic	N	NEG	M	1
45	82	F	Stomach	E	WT	WT	WT	WT	*SDHA* Exon10 p.R465Q (c.1394G>A) homozygous somatic	N	NEG	M	3
46	28	M	Stomach	E	WT	WT	WT	WT	*SDHB* Exon6 p.M213R (c.638 T>G) homozygous somatic	N	NEG	M	1
47	49	F	Stomach	E	WT	WT	WT	WT	*SDHC* Exon4 p.G75D (c. 224G>A) heterozygous somatic	N	NEG	U	3
48	27	F	Stomach	M	WT	WT	WT	WT	*SDHA* Exon9 p.G419R (c. 1255G>A) heterozygous (germline not tested)	N	NEG	M	NA

^a^*S* spindle cell, *E* epithelioid, *M* mixed spindle cell and epithelioid. ^b^Wild type. ^c^*NA* not assessed. ^d^*Y* yes. *N* no. ^e^*M* methylated. *U* unmethylated. ^f^*POS* positive. *NEG* negative. ^g^*NOS* not otherwise specified. ^h^Previously published [22]. ^i^Previously published [27]. ^j^Previously published [26]. ^k^DNA analyzed in one of the two tumors available; the DNA from the other one was degraded

### SDH analysis

SDHB IHC was performed on all *KIT* and *PDGFRA*-WT cases, irrespective of site or morphology. Although GISTs depending on molecular triggers involving genes other than *SDHx* have been consistently proved SDHB-positive [28], we investigated for SDHB expression fifteen of such tumors in our series as a control. Mouse monoclonal antibody to SDHB 21A11 (ABCAM, Cambridge, MA, 1:1000) was employed. The Leica BondMax autostainer (Leica Microsystems, Bannockburn, IL) was employed utilizing the BondMax avidin biotin-free polymer-based detection system preceded by heat-induced epitope retrieval with Leica retrieval solution (alkaline buffer), using diaminobenzine as the chromogen. Only slides with positive internal control (smooth muscle, endothelial, epithelial, or lymphoid cells) were considered for analysis.

Genetic analysis of *SDH* subunits was performed in SDHB-negative cases as follows. The exons of the subunits of SDH complex were sequenced on tumor and normal tissue using the Sanger sequencing method on ABI 310 Genetic Analyzer (Applied Biosystems). DNA was extracted from tumor and normal FFPE specimens by the QIAmp DNA Micro kit (Qiagen, Milan, Italy) in accordance with manufacturer’s recommendations. Briefly, FFPE slices (three 10-μm-thick slices for each sample) were digested overnight at 56 °C in ATL buffer with the addition of proteinase K (Qiagen). DNA extraction was then continued with QIAamp DNA micro kit (Qiagen). Each exon was amplified with polymerase chain reaction (PCR) amplification using specific primer pairs, as previously reported [29]. PCR was carried out in a total volume of 25 μl consisting in 20 ng of DNA, 10 × PCR buffer, MgCl2, dNTP, primers (10 pM each), and 1 U FastStart DNA Taq polymerase (Roche). PCR conditions were an initial denaturation of 95 °C for 5 min, followed by 40 cycles of 95 °C for 30 s, 52–64 °C for 30 s, and 72 °C for 30 s. PCR products were purified with the Qiaquick PCR purification kit (Qiagen) and sequenced on both strands using the Big Dye Terminator v1.1 Cycle Sequencing kit (Applied Biosystems).

### MGMT analysis

DNA extraction and bisulfite modification were performed on three 10-μm slides from paraffin-embedded tissues, as previously described [30]. The pathologic areas selected for DNA extraction contained at least 70% disease-specific cells. The methylation status of the GpG islands in the promoter region of *MGMT* was determined as described elsewhere [31]. Briefly, bisulfite-modified DNA (100–200 ng) was amplified in a mixture containing 1 × PCR buffer (20-mM Tris [pH 8.3], 50-mM KCl, 1.5-mM MgCl2), deoxynucleotide triphosphates (0.2 mM each), primers (20 pM each), and 0.75 U GoTaq Hot Start polymerase (Promega, Madison, Wis) in a final volume of 25 μl. PCR conditions were an initial denaturation of 95 °C for 8 min, followed by 35 cycles of 95 °C for 60 s, 60 °C for 60 s, and 72 °C for 60 s. PCR products were electrophoresed in a 2.5% agarose gel, stained with ethidium bromide, and visualized under ultraviolet illumination (93 and 81 bp were the lengths of the unmethylated and methylathed bands, respectively). Methylation-specific PCR (MS-PCR) analysis was performed in duplicate for all samples. Normal lymphocyte DNA supermethylated with SssI methyltransferase (New England Biolabs, Beverly, Mass) and treated with bisulfite was used as the unmethylated and methylated control, water as a negative control, and untreated DNA as internal PCR control.

For MGMT immunohistochemistry, mouse monoclonal antibody to MGMT MT 23.2 (Thermo Fisher, Waltham, MA, 1:2000) was employed. The Dako autostainer link 48 was employed utilizing the EnVision™ FLEX+ detection system (Dako, Glostrup, Denmark) preceded by heat-induced epitope retrieval (EDTA 120°, 10 min). Only slides with positive internal control (endothelial, lymphoid, epithelial, or smooth muscle cells) were considered for analysis. MGMT expression was scored by two pathologists, blinded to tumor methylation status, molecular, and clinical data, using a three-tiered scale as previously reported (scores 1, 2, and 3 for < 10%, 10 to 49%, and > 50% tumor cells featuring intensely and uniformly stained nuclei, respectively) [32].

### Statistical analysis

Two-tailed Fisher’s exact tests were performed to compare pairs of data sets using Statistica 12 software (Statsoft Inc., Tulsa, OK).

## Results

### SDH analysis

To identify functional loss of the SDH complex in GISTs, we performed SHDB immunohistochemistry [33]. Nine GISTs were SDHB-negative. All of them were gastric, non-NF1-related, at-least-in-part epithelioid (see Additional file 1: Figure S1, which shows a typical SDH-negative GIST from our series) and WT for *KIT*, *PDGFRA*, *KRAS*, and *BRAF*. All these GISTs harbored mutations in one of the subunits of *SDH*, namely, *SDHA* in seven cases, *SDHB* in one case, and *SDHC* in one case. A somatic second hit flanked a germline mutation in three cases; in five tumors, the genetic alterations were exclusively somatic: in two cases, homozygous (likely due to loss of heterozygosis), in one case compound heterozygous, and in two cases heterozygous; in one case, the germline *SDH* status could not be investigated (Table 1 and Additional file 2: Figure S2, which shows chromatograms of the *SDHx* mutations found in cases 42–48 of our series).

### MGMT analysis

**Fig. 1:**
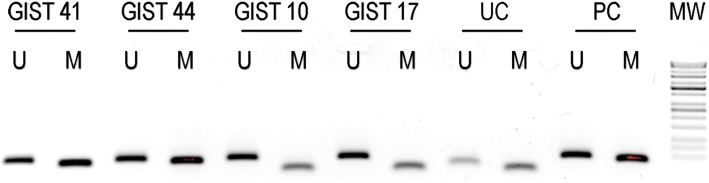
Methylation status of *MGMT* promoter gene (MS-PCR) in four examples of GIST. Lanes containing PCR products derived from unmethylated and methylated alleles are marked U and M, respectively (with a length of 93 and 81 bp, in that order). GISTs 41 and 44 show a hypermethylated *MGMT* promoter gene; the same gene is unmethylated in GISTs 10 and 17 (the bands present in the M lanes of these two GISTs and in both lanes of UC are primer dimers). UC untreated control DNA, PC positive control DNA, MW molecular weight marks (100-bp ladder)

To ascertain whether *MGMT* promoter CpG islands were methylated in GISTs, and whether *MGMT* methylation pattern varied among the pathogenetically heterogeneous GIST subgroups of our series, we analyzed *MGMT* by MS-PCR. *MGMT* was methylated in only 1 out of 24 (4%) *KIT* or *PDGFRA-*mutant GISTs (namely, an exon-11-*KIT*-mutant case), against 11 *MGMT*-methylated cases of the remaining 24 (46%) *KIT*/*PDGFRA*-WT GISTs (*p* = 0.002, Fisher exact test). *KRAS* and *BRAF* were WT in all the evaluated cases of our series, including all the *KIT*/*PDGFRA* WT tumors. Six out of 9 (67%) and 6 out of 39 (15%) cases revealed *MGMT*-methylated among SDHB-deficient and SDHB-proficient GISTs, respectively (*p* = 0.004, Fisher’s exact test). *MGMT* was methylated in one out of the five NF1-related GISTs. Figure 1 is a representative of the MS-PCR results for *MGMT* promoter in some GISTs of our series. Data are resumed in Table 1.

**Fig. 2:**
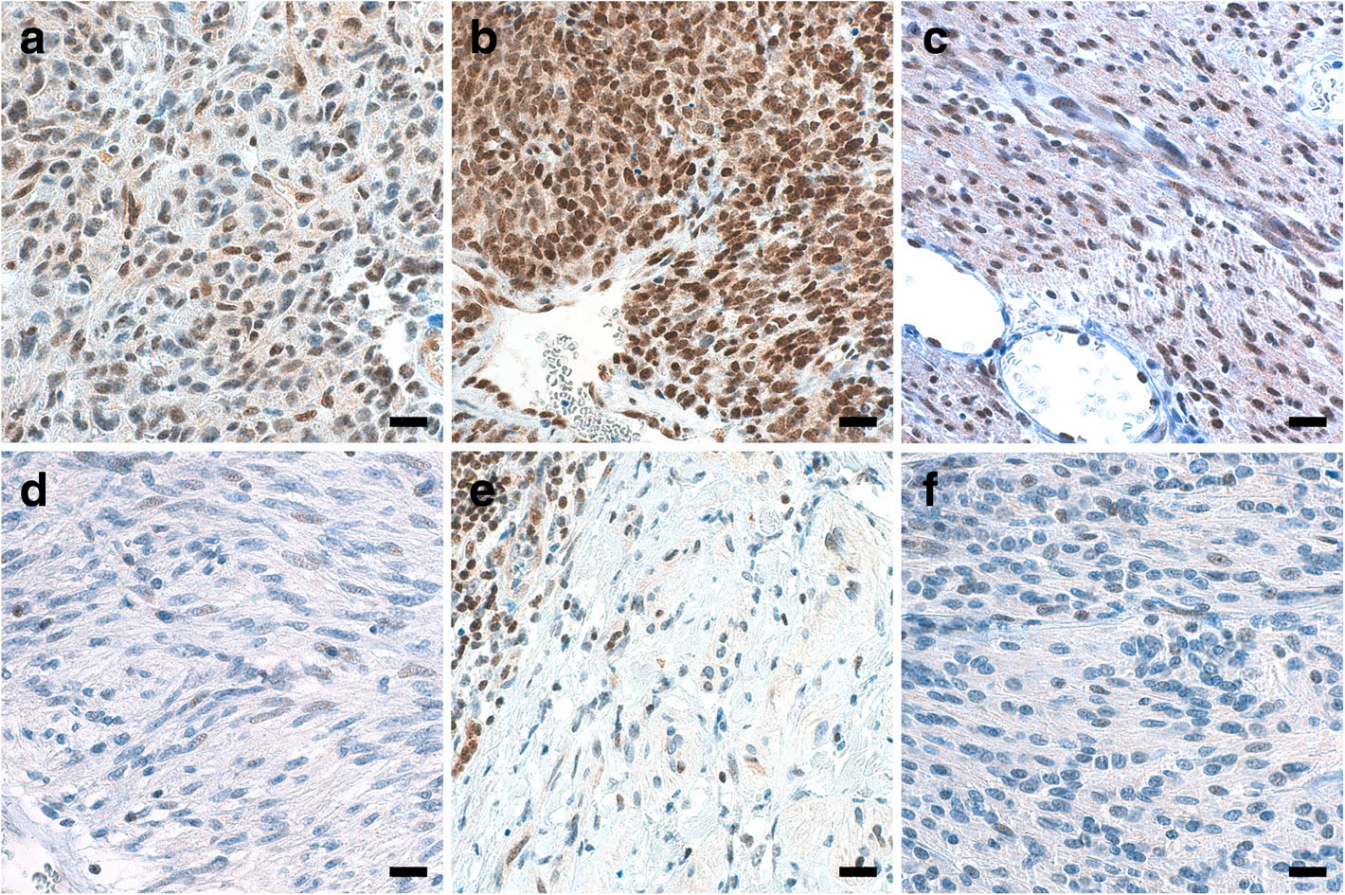
MGMT protein expression assessed by IHC in six examples of GIST. At MGMT IHC, *MGMT*-unmethylated GISTs 43 (**a**), 26 (**b**), and 27 (**c**) featured nuclear staining in the majority of cells (score 3), unlike *MGMT*-methylated GISTs 32 (**d**), 31 (**e**), and 44 (**f**) (score 1). Endothelial and lymphoid cells (the periphery of a lymphoid aggregate is evident in **e**, top left) act as internal positive controls. (Scale bars: 20 μm)

To assess the impact of *MGMT* methylation on MGMT expression, we performed MGMT immunohistochemistry in half of GISTs of our series. The overall correlation between *MGMT* promoter methylation and MGMT immunohistochemical expression (score 1 vs. scores 2–3) was good (*p* = 0.001, Fisher’s exact test), although a minority of tumors showed either a high-MGMT IHC score in the presence of *MGMT* methylation or the reverse condition (Table 1, Fig. 2).

## Discussion

We herein assessed *MGMT* methylation status in a population of GISTs heterogeneous with respect to pathogenesis. Our results show that a subset of GISTs WT for *KIT* and *PDGFRA*, including their largest pathogenetically characterized subpopulation, i.e., the SDH-deficient ones, is significantly enriched in *MGMT*-methylated cases, with SDH-deficient GISTs featuring the highest prevalence of this tumor variant. Thus, these subsets of GISTs, characterized by a problematic clinical management in case of malignancy because of their usual resistance to imatinib and variable response to other drugs, appear potentially predisposed to respond to alkylating agents.

GISTs WT for *KIT* and *PDGFRA* constitute a pathogenetically heterogeneous tumor group accounting for about 10–15% of GISTs [1]. Taken together, these GISTs are characterized by an overall indolent behavior [3, 4]. However, malignant cases may occur, often accompanied by unresponsiveness to the commonly used TKI therapies [5].

SDH-deficient GISTs, characterized by negativity at SDHB immunostaining, constitute the largest pathogenetically characterized subgroup among cases WT for *KIT* and *PDGFRA*. Their pathogenesis basically follows the classic tumor-suppressor gene model, with both alleles of one of the four *SDH* subunits inactivated in the tumor cells, either on a mutational (often with a germline component) or epigenetic (*SDHC* hypermethylation) base, possibly presenting in syndromic settings (Carney triad or Carney-Stratakis syndromes) [3, 12, 28, 34]. Their progression can be extremely slow, with reported time lapses between primary tumor and metastasis as long as 42 years [33], and their stage and clinical behavior are often incoherent, with frequent prolonged survival in the presence of metastases. These features, likely caused by the metabolic disadvantage due to SDH deficiency, can make the attribution of disease stability to a drug effect problematic [7]. Nevertheless, SDH-deficient GIST can behave aggressively, with an overall mortality probably higher than 15%, with cases fatal in a few years [28].

Imatinib, a TKI acting on several KIT and PDGFRA isoforms, proved ineffective in SDH-deficient GISTs [28], coherently with SDH deficiency being a trigger independent of KIT/PDGFRA. No mature data concerning the use of sunitinib in these neoplasms are available, although a clinical benefit due to an anti-angiogenic mechanism may occur. Of note, the rarity of SDH-deficient GISTs makes investigations on pure populations of such tumors very difficult. Consequently, clues for establishing an effective chemotherapy for this GIST subset are often inferred from studies on *KIT/PDGFRA*-WT cases, which are enriched in SDH-deficient tumors. Following these premises, drugs of potential interest for treating the latter neoplasms, in part represented by experimental molecules based on their biology, include linsitinib, PI3K/AKT/mTOR inhibitors, nilotinib, sorafenib, heat-shock protein inhibitors, chemicals targeting hypoxia-inducible factor-1α, and demethylating agents such as decitabine [14, 35–39]. Nevertheless, we presently do not dispose of a reliably effective drug for treating SDH-deficient GISTs, with the possible exception of regorafenib, recently reported to induce objective responses and durable benefit [6].

*MGMT* inactivation through gene methylation allows the effective employment of alkylating agents in heterogeneous human tumors [8–11]. SDH-deficient GISTs display DNA methylation (both in the presence of *SDHx* mutations or not) which, although overall pervasive, is not randomly distributed [12–14]. We therefore studied *MGMT* methylation in the SDH-deficient GIST subgroup, as a premise to possible investigations on the employment of alkylating agents for their treatment.

We identified nine SDH-deficient GISTs, as assessed by SDHB protein loss, all of which harboring mutations in one of the *SDH* subunits (Table 1). Epigenetic inactivating mechanisms, such as allelic hemimethylation, can be hypothesized to explain the loss of SDHB protein in the three tumors of our series lacking a detectable second hit, bearing a heterozygous *SHDx* mutation; in fact, combined heterozygous mutation and hemimethylation have been reported in *SDHx* (namely, *SDHC*) in some GISTs; moreover, *SDHA*-mutant GISTs which, despite the absence of a detectable second hit, nevertheless featured loss of SDHB expression have also been signaled [14]. Of note, our finding of five cases lacking germline *SDHx* mutations out of 8 *SDHx*-mutant SDH-deficient ones whose germline could be investigated could possibly support a relatively common occurrence of this GIST type in the absence of a syndromic predisposition.

In our series, all *MGMT*-methylated cases but one were detected in the pathogenetically heterogeneous group of GIST WT for *KIT* and *PDGFRA*, and SDH-deficient GISTs were significantly enriched in *MGMT*-methylated cases; noticeably, with the limitations due to the small number of cases considered, SDH-deficient GISTs featured the highest prevalence of *MGMT* methylation (6/9, 67%) when compared to the other pathogenetically characterized GIST subgroups of our series (0/10, 0%, of *PDGFRA*-mutant GISTs; 1/15, 7%, of *KIT*-mutant GISTs; 1/5, 20%, of NF1-associated GISTs; Table 1). Additionally, we found an overall good correlation between *MGMT* methylation and MGMT protein expression, as assessed by IHC (the discrepancy found in a minority of cases could depend on DNA methylation levels not enough to inhibit protein expression on the one hand, or on mechanisms affecting protein expression alternative to DNA methylation, such as histone modifications or post-transcriptional regulation, on the other [40]). Thus, our results support the potential predisposition to respond to alkylating agents of a subset of the hitherto globally chemorefractory *KIT/PDGFRA* WT GISTs, including their largest pathogenetically characterized subtype: SDH-deficient ones.

We do not know whether the *MGMT* methylation we observed in *KIT/PDGFRA*-WT GISTs, including those SDH-deficient, is due to a mechanism specifically targeting *MGMT* or not (of note, this issue would not affect the potential therapeutic implications of the event). The DNA methylation described in SDH-deficient GISTs, caused by the impairment of the conversion of 5-methylcytosine to 5-hydroxymethylcytosine (required for DNA demethylation) due to the inhibition of TET DNA-hydroxylases secondary to succinate accumulation [3], could non-specifically contribute to the enrichment in *MGMT*-methylated cases we found among SDH-deficient GISTs. Nevertheless, since, as noted above, DNA methylation in these latter tumors is not randomly distributed [12–14], hitherto unknown gene-specific mechanisms regulating DNA methylation, possibly similar to that exerted by *HOTAIR* in some GISTs [41], are bound to flank the non-specific DNA methylation machinery described in SDH-deficient GISTs, and could affect *MGMT* also. Conversely, to the best of our knowledge, neither relatively high-DNA methylation levels, nor DNA methylation mechanisms, have been described in *KIT/PDGFRA*-WT, SDH-competent GISTs, explaining the significant *MGMT* methylation we found in these tumors.

The cases of our series were arbitrarily assorted with the aim of investigating *MGMT* methylation in GIST subgroups differing in molecular trigger; consequently, rare GIST types such as SDH-deficient and *NF1*-related ones are overrepresented with respect to population-based series. Nevertheless, considering the known epidemiology of GISTs [1], our finding of *MGMT* methylation substantially restricted to almost half of the investigated *KIT/PDGFRA* WT cases, even though these also were arbitrarily assorted in our series, supports a rare occurrence of this condition in GISTs, probably accounting for < 5–10% of cases. To the best of our knowledge, only four papers have reported on *MGMT* methylation or expression in GISTs, employing heterogeneous methods. Two of them, both employing MS-specific PCR, report a relatively high fraction of *MGMT*-methylated GISTs, potentially challenging the concept that *MGMT* methylation is a relatively rare event in these tumors [15–17, 20]. However, one of these papers dealt exclusively with gastric cases [15], which are expectedly enriched in SDH-deficient GISTs, albeit presumably not at a level to justify per se the detected prevalence of *MGMT*-methylation (of note, SDH status was not investigated, coherently with the epoch of the study, which preceded the discovery of the role of SDH in GIST pathogenesis [42]). The paper by Saito and co-workers [16] overtly contrasts with our findings in terms of expected low prevalence of *MGMT*-methylated GISTs; nonetheless, all three of the three (100%) reported cases WT for both *KIT* and *PDGFRA* featured a methylated *MGMT*. Noticeably, none of these works concerning *MGMT* methylation in GISTs was population-based.

Published data on the effectiveness of alkylating agents temozolomide and carmustine in GIST treatment, although apparently discouraging, do not stand definitively against a possible efficacy of alkylating agents restricted to a subset of the uncommon *KIT/PDGFRA* WT GISTs, including SDH-deficient ones. In fact, these results are based on a limited number of GIST patients recruited in past trials on sarcomas, without either selecting the genotype or investigating the SDH status of the tumors [18–20]. The latter event is again coherent with the epoch of the referred studies. Moreover, MGMT status was studied only in two of these tumors, significantly revealing spontaneous activity and, although O^6^-benzylguanine was administered with the aim of inhibiting this enzyme, the achievement of such an effect was not verified [20]. Finally, Choi response criteria, the most sensitive method for detecting GIST response to drugs [21], were not employed, once more coherently with the epoch of the referred papers. Thus, a possible effect of alkylating agents on a predisposed fraction of GISTs could have so far escaped detection since (1) we herein prove that *MGMT* methylation is substantially restricted to rare subsets of these tumors, implying the possibility that a few (or even none) of the limited number of GIST patients enrolled in the abovementioned trials could respond to the administered therapy, and (2) a possible response of some GISTs, bearing an inactivated MGMT, could have escaped detection, given the missed adoption of Choi criteria. This scenario could have led to erroneously abort further studies on alkylating agents in GISTs. As confirmation of our hypothesis, a phase 2 trial investigating a response to the drug temozolomide in metastatic SDH-deficient GIST to improve patient outcomes was recently launched and is ongoing (ClinicalTrials.gov/NCT03556384).

## Conclusions

A subset of *KIT/PDGFRA*-WT GISTs, including their largest pathogenetically characterized subgroup (i.e., SDH-deficient GISTs), is preferentially *MGMT*-methylated. This finding fosters a reappraisal of alkylating agents for treating malignant cases occurring among these overall chemorefractory tumors.

## Additional files

Additional file 1: **Figure S1.** Microphotograph showing a SDH-deficient GIST (case 43). (a) Tumor consisted of sheets of epithelioid cells with eosinophilic cytoplasm (scale bar: 15 μm). (b) Tumor cells lack cytoplasmic SDHB granular positivity, retained in non-neoplastic cells (notice the cytoplasmic labeling of smooth muscle cells, both in gastric muscularis propria—top left—and in a blood vessel wall—center—, or of scattered tumor infiltrating leukocytes and plasma cells) (scale bar: 15 μm). (TIF 16742 kb)

Additional file 2: **Figure S2.** Chromatogram showing the *SDHx* mutations found in GISTs (T) and in normal tissue (N) in cases 42–48. Case 42: heterozygous *SDHA* exon 5 mutation in normal tissue and homozygous in GIST. Case 43: somatic heterozygous *SDHA* exons 1 and 13 mutations in GIST. Case 44: somatic heterozygous *SDHA* exon 9 mutation in GIST. Case 45: somatic homozygous *SDHA* exon 10 mutation in GIST. Case 46: somatic homozygous *SDHB* exon 6 mutation in GIST. Case 47: somatic heterozygous *SDHC* exon 4 mutation in GIST. Case 48: heterozygous *SDHA* exon 9 mutation in GIST (germline not tested). (*SDHx* mutations found in cases 40 and 41 have been previously reported—see ref. [26] of the main text). (PPTX 1802 kb)

## Abbreviations

GIST: Gastrointestinal stromal tumor
MGMT: O^6^-methylguanine DNA methyltransferase
PDGFRA: Platelet-derived growth factor receptor alpha
SDH: Succinate dehydrogenase
