# Supplementary figures and images for: Preferential MGMT methylation could predispose a subset of KIT/PDGFRA-WT GISTs, including SDH-deficient ones, to respond to alkylating agents
Source: Clin Epigenetics. 2019 Jan 7;11:2. doi: 10.1186/s13148-018-0594-9 (PMC6322231; doi:10.1186/s13148-018-0594-9)

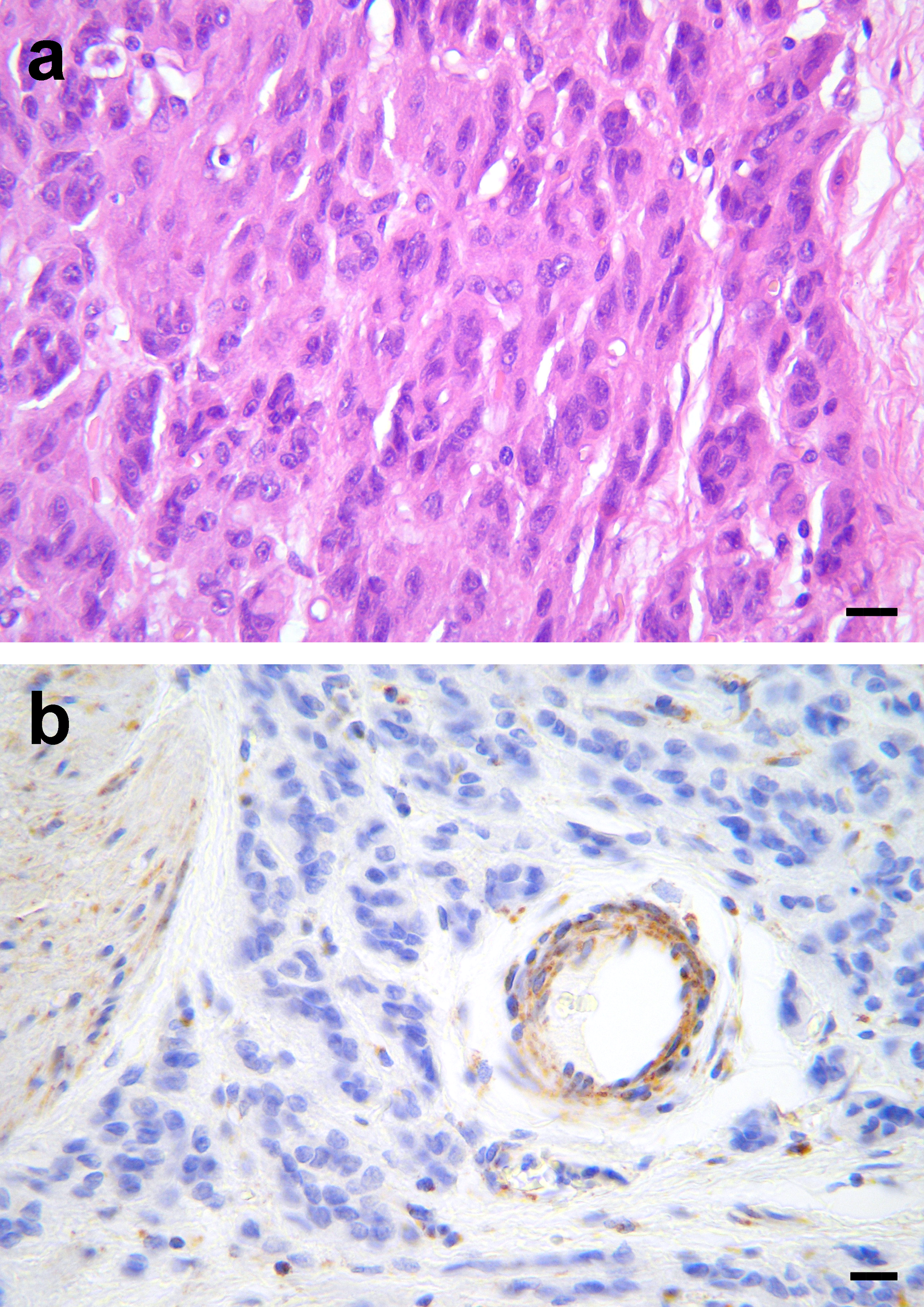

Supplement: Supplementary file 1 — Figure S1. Microphotograph showing a SDH-deficient GIST (case 43). (a) Tumor consisted of sheets of epithelioid cells with eosinophilic cytoplasm (scale bar: 15 μm). (b) Tumor cells lack cytoplasmic SDHB granular positivity, retained in non-neoplastic cells (notice the cytoplasmic labeling of smooth muscle cells, both in gastric muscularis propria—top left—and in a blood vessel wall—center—, or of scattered tumor infiltrating leukocytes and plasma cells) (scale bar: 15 μm). (TIF 16742 kb) [file 13148_2018_594_MOESM1_ESM.tif]
