# Supplementary material for: Preferential MGMT methylation could predispose a subset of KIT/PDGFRA-WT GISTs, including SDH-deficient ones, to respond to alkylating agents
Source: Clin Epigenetics. 2019 Jan 7;11:2. doi: 10.1186/s13148-018-0594-9 (PMC6322231; doi:10.1186/s13148-018-0594-9)

## Slide 1
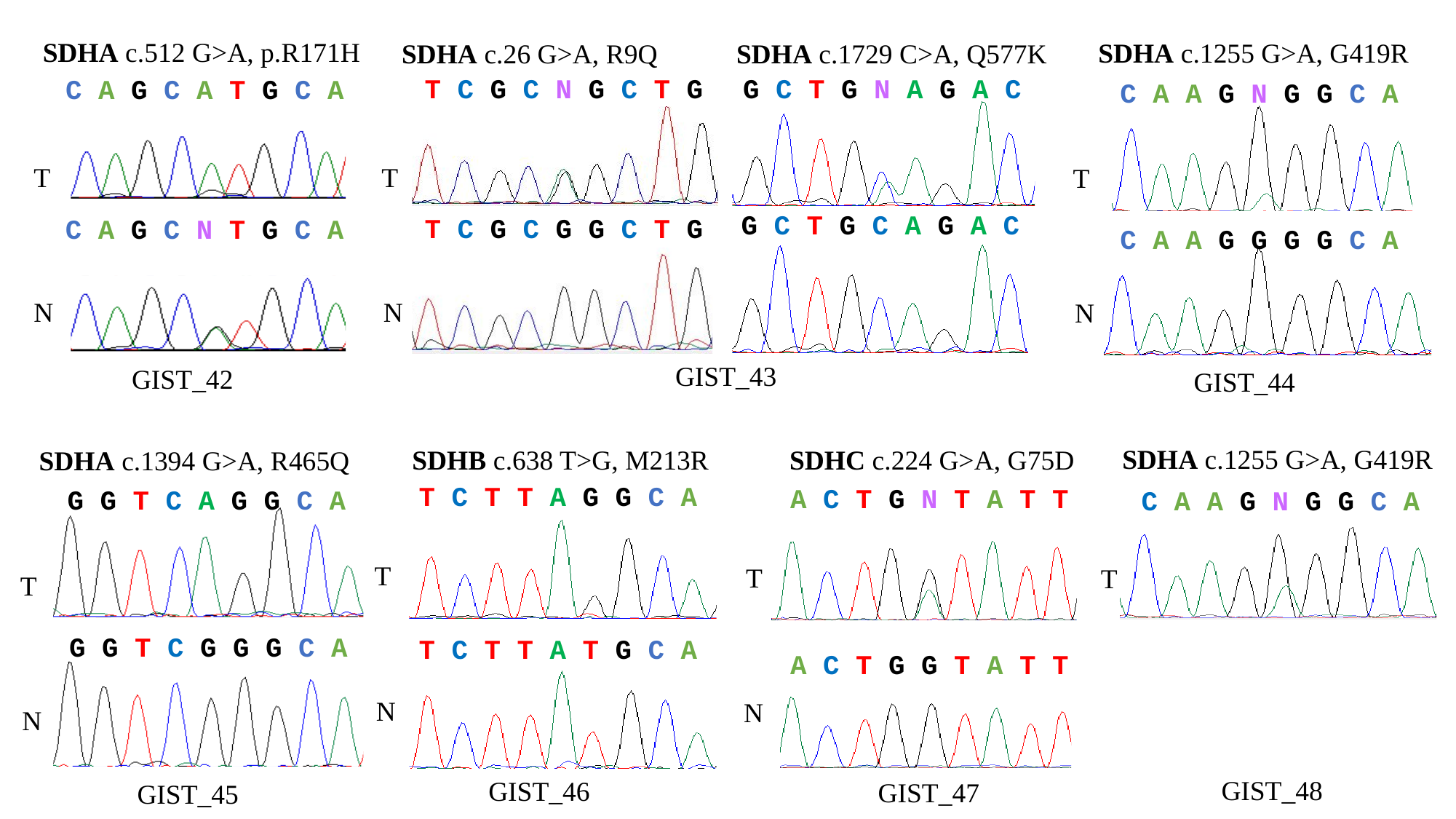

SDHA c.512 G>A, p.R171H
SDHA c.1255 G>A, G419R
C A A G N G G C A
T
C A A G G G G C A
N
GIST_44
SDHA c.26 G>A, R9Q
SDHA c.1729 C>A, Q577K
T C G C N G C T G
T C G C G G C T G
G C T G N A G A C
G C T G C A G A C
T
N
GIST_43
C A G C A T G C A
T
C A G C N T G C A
N
GIST_42
SDHA c.1255 G>A, G419R
SDHB c.638 T>G, M213R
SDHC c.224 G>A, G75D
SDHA c.1394 G>A, R465Q
G G T C A G G C A
T
G G T C G G G C A
N
GIST_45
T C T T A G G C A
T
T C T T A T G C A
N
A C T G N T A T T
C A A G N G G C A
T
T
A C T G G T A T T
N
GIST_48
GIST_46
GIST_47

## Slide 2
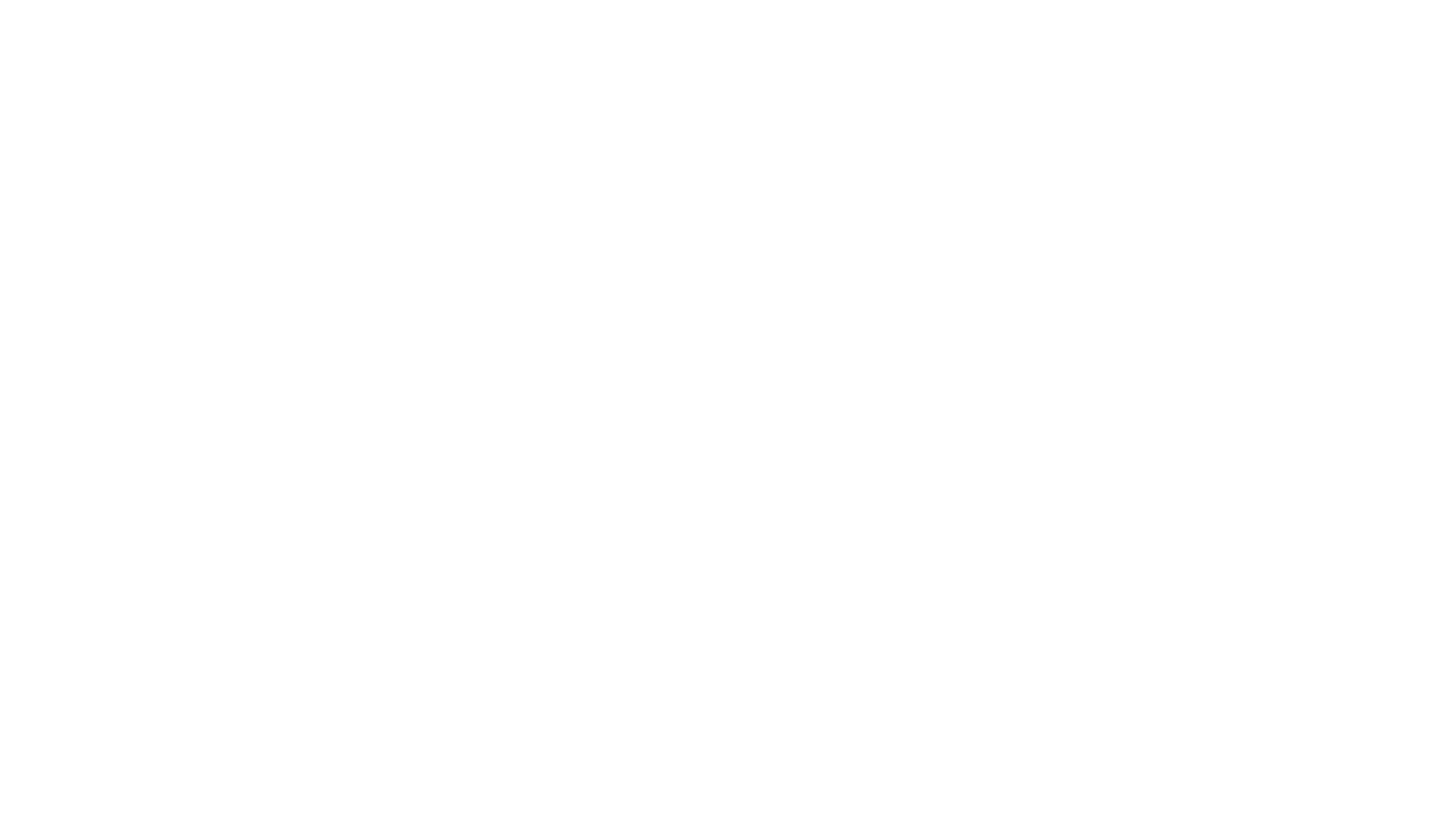

Supplement: Supplementary file 2 — Figure S2. Chromatogram showing the SDHx mutations found in GISTs (T) and in normal tissue (N) in cases 42–48. Case 42: heterozygous SDHA exon 5 mutation in normal tissue and homozygous in GIST. Case 43: somatic heterozygous SDHA exons 1 and 13 mutations in GIST. Case 44: somatic heterozygous SDHA exon 9 mutation in GIST. Case 45: somatic homozygous SDHA exon 10 mutation in GIST. Case 46: somatic homozygous SDHB exon 6 mutation in GIST. Case 47: somatic heterozygous SDHC exon 4 mutation in GIST. Case 48: heterozygous SDHA exon 9 mutation in GIST (germline not tested). (SDHx mutations found in cases 40 and 41 have been previously reported—see ref. [26] of the main text). (PPTX 1802 kb) [file 13148_2018_594_MOESM2_ESM.pptx]
